# Inverse T-shaped sternotomy as novel thoracoplasty for severe chest deformation and tracheal stenosis

**DOI:** 10.1186/s40792-021-01275-8

**Published:** 2021-08-26

**Authors:** Hirofumi Tomita, Akihiro Shimotakahara, Naoki Shimojima, Hideo Ishihama, Miki Ishikawa, Yuki Mizuno, Makoto Hashimoto, Ayano Tsukizaki, Kazuaki Miyaguni, Seiichi Hirobe

**Affiliations:** Department of Surgery, Tokyo Metropolitan Children’s Medical Center, 2-8-29 Musashidai, Fuchu, Tokyo, 183-8561 Japan

**Keywords:** Severe motor and intellectual disabilities, Chest deformation, Tracheal stenosis, Brachiocephalic artery transection, Thoracoplasty

## Abstract

**Background:**

Patients with severe motor and intellectual disabilities often suffer from tracheal stenosis due to chest deformation and brachiocephalic artery compression, which sometimes leads to serious complications, such as dying spell and tracheobrachiocephalic artery fistula. We herein described our experience of performing a novel and simple thoracoplastic procedure combined with brachiocephalic artery transection in two patients with severe chest deformation and tracheal stenosis.

**Case presentation:**

The patients were a 12-year-old female with cerebral palsy due to periventricular leukomalacia and a 21-year-old male with subacute sclerosing panencephalitis stage IV in the Jabbour classification following a laryngotracheal separation. Both patients showed severe chest deformation and symptoms of airway stenosis resulting in dying spells. The sternum was laterally transected between the manubrium and the sternal body, and a manubriotomy was performed longitudinally, ending with an inverse T-shaped sternotomy. Since the clavicle and the first rib remained attached to the halves of the divided manubrium, the sternum was allowed to be left open, resulting in improvement of the mediastinal narrowing and tracheal stenosis. Postoperative computed tomography (CT) showed that the distance between the halves of the manubrium was maintained at 10–11 mm, and that the mediastinal narrowing in both patients improved; the sternocervical spine distance increased from 20 mm to 22  and 13 mm to 16 mm, respectively. The patients’ tracheal stenosis below the sternal end of the clavicle and the manubrium and respiratory symptoms improved, and the patients are currently at home in a stable condition with no chest fragility and no upper limb movement disorder 1 year after surgery.

**Conclusions:**

Our observations suggested that the inverse T-shaped sternotomy combined with brachiocephalic artery transection may relieve symptoms of tracheal stenosis due to severe chest deformation in patients with severe motor and intellectual disabilities.

## Background

**Fig. 1:**
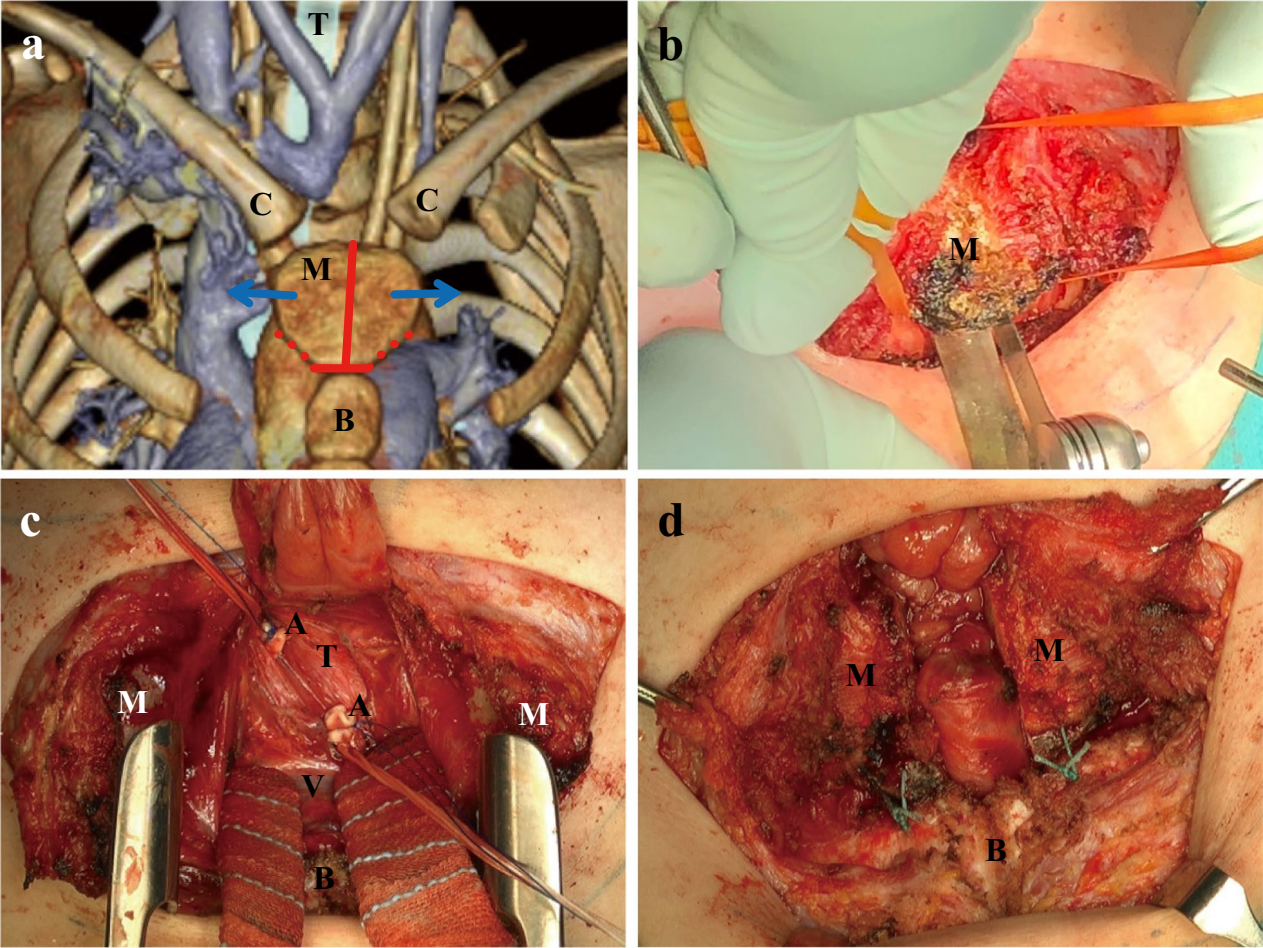
Imaging and intraoperative photographs in Patient 1. **a** Concept of the inverse T-shaped sternotomy on three-dimensional computed tomography (CT) imaging. Red and dotted lines indicate the lines of the sternotomy and the line of detachment between the second costal cartilage and the manubrium, respectively. **b** Manubriotomy from the caudal side using an oscillator. **c** Brachiocephalic artery transection. Note that the trachea is compressed between the transected artery. **d** Fixation of the halves of the divided manubrium to the bilateral second costal cartilage. * A* brachiocephalic artery;* B* sternal body;* C* sternal end of the clavicle;* M* manubrium sterni;* T* trachea;* V* brachiocephalic vein

Patients with severe motor and intellectual disabilities commonly suffer from muscle tone abnormalities, such as spasticity and hypotonia resulting in scoliosis and chest deformation. In some cases, severe chest deformation and brachiocephalic artery compression cause tracheal stenosis, leading to tracheomalacia-like symptoms, such as stridor, dyspnea, and life-threatening anoxic spells (dying spell) [1]. Furthermore, patients with tracheal stenosis due to brachiocephalic artery compression and tracheostomy have a high risk of intratracheal granulation and tracheobrachiocephalic artery fistula due to tracheal tube stimulation [2]. A distance < 20 mm between the posterior surface of the sternum and the anterior surface of the cervical spine (sternocervical spine distance) is considered a risk factor of tracheobrachiocephalic artery fistula development [3]. To date, few reports have described thoracoplasty for severe chest deformation and tracheal stenosis. We herein described a novel and simple thoracoplastic procedure, the inverse T-shaped sternotomy (Fig. 1a), combined with brachiocephalic artery transection.

### Surgical technique

A transverse skin incision was made along Langer's line at the manubrium sterni. The subcutaneous layer was widely detached from the fascia, and a median, longitudinal incision was made in the fascia and periosteum to expose the sternum. The fascial incision was transversely widened at the height of the sternal angle, and the first intercostal space and the attachment between the second costal cartilage and the manubrium were incised. Thereafter, the sternum was transected between the manubrium and the body of the sternum using an electric knife and Luer forceps. The dorsal and cranial sides of the manubrium were exposed, and a manubriotomy was performed longitudinally from the caudal side using an oscillator (Fig. 1b). After transecting the brachiocephalic artery (Fig. 1c), the separated halves of the manubrium remained about 1 cm apart. The halves were affixed to the bilateral second costal cartilage using 1–0 non-absorbable sutures (Fig. 1d). Closed suction drains were placed, one in the mediastinum, the other subcutaneously, and the fascia and skin incisions were closed. The operation was performed after confirming that the circle of Willis was intact on an imaging study. Computed tomography (CT) imaging was done at postoperative 3 months to evaluate the extent of the space below the manubrium and tracheal stenosis. Written informed consent was obtained from the patients’ guardians for the procedure.

## Case presentations

### Patient 1

**Fig. 2:**
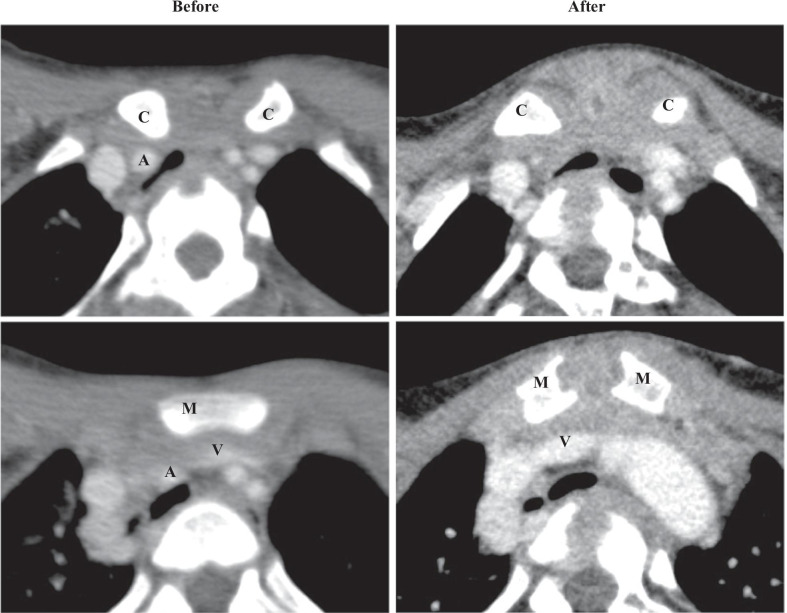
CT imaging before and after transection of the brachiocephalic artery and thoracoplasty in Patient 1.* A* brachiocephalic artery;* C* sternal end of the clavicle;* M* manubrium sterni;* V* brachiocephalic vein

Patient 1 was a 12-year-old female with cerebral palsy due to periventricular leukomalacia. She showed increased muscle tone, significant opisthotonus, and symptoms of airway stenosis sometimes resulting in dying spells. The trachea was extensively flattened along with mediastinal narrowing (the sternocervical spine distance was 20 mm), and tracheal stenosis was particularly prominent on the dorsal side of the sternal end of the right clavicle and brachiocephalic artery. The brachiocephalic vein was obstructed, and large collateral circulation was observed in the anterior neck. The operation time was 4 h 37 min, and the amount of bleeding was 10 ml. Soon after the operation, dying spell due to malacia of the tracheal bifurcation was observed during tracheal intubation management. However, the symptoms of airway stenosis improved after extubation, and the dying spell did not recur. Figure 2 shows the pre- and post-operative CT imaging findings. The convexity of the trachea was restored postoperatively, ameliorating the tracheal stenosis. The distance between the halves of the manubrium was maintained at 11 mm, and the sternocervical spine distance was increased from 20 to 22 mm. The prism of the sternal end of the clavicle rotated outward, relieving the tracheal compression between the clavicle and vertebrae. Moreover, the cut surface of the manubrium faced slightly upward like a drawbridge, and the patency of the brachiocephalic vein was restored, suggesting improvement of the mediastinal narrowing. The patient is currently at home in stable condition with no chest fragility and no upper limb movement disorder 1 year after surgery.

### Patient 2

**Fig. 3:**
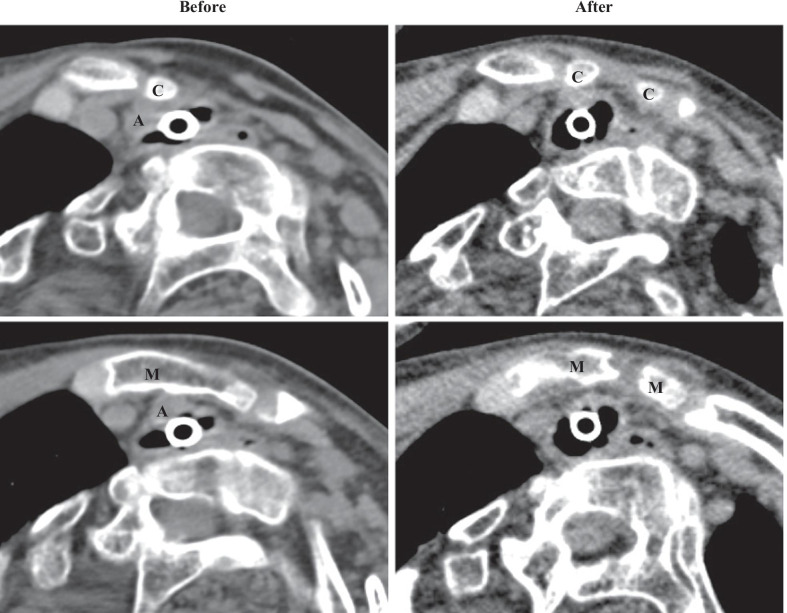
Computed tomography imaging before and after transection of the brachiocephalic artery and thoracoplasty in Patient 2. * A* brachiocephalic artery;* C* sternal end of the clavicle;* M* manubrium sterni

A 21-year-old, male patient with subacute sclerosing panencephalitis stage IV in the Jabbour classification underwent laryngotracheal separation at age 20 years. He was referred to us for marked intratracheal granulation and frequent dying spells. The space between the sternum and vertebrae showed narrowing (the sternocervical spine distance was 13 mm) due to severe scoliosis, and he was assumed to have a high risk of developing a tracheobrachiocephalic fistula owing to tracheostomy tube stimulation (Fig. 3, left column). The operation time was 3 h 50 min, and the amount of bleeding was 34 ml. Postoperatively, no dying spells occurred, the intratracheal granulation gradually decreased, and home management became possible. Postoperative CT showed improvement of the tracheal stenosis below the sternal end of the clavicle and the manubrium, the distance between the halves of the divided manubrium remained 10 mm (Fig. 3, right column), and the sternocervical spine distance increased from 13 to 16 mm. At postoperative 1 year, the patient is at home in stable condition with no chest fragility and no upper limb movement disorder.

## Discussion

Brachiocephalic artery transection with or without revascularization is performed for respiratory symptoms due to tracheal stenosis caused by brachiocephalic artery compression in patients with neurological or neuromuscular disorders [1]. Because tracheobrachiocephalic artery fistula development is associated with an extremely poor prognosis, evaluating the risk factors in a patient and preventing its development are essential before performing a tracheostomy [2]. The basic surgical approach in a brachiocephalic artery transection is a total or partial median sternotomy [1, 4]. Various alternatives, such as oblique partial manubriotomy [5], the suprasternal approach [3, 6], and the left anterior extrapleural approach [7], were reportedly effective in minimizing invasiveness and the risk of wound contamination. However, these alternatives are limited by various types of chest deformation and anatomical displacement of the brachiocephalic artery in individual cases [1].

Because tracheal stenosis in patients with severe motor and intellectual disabilities is caused by chest deformation, thoracoplasty holds out the promise of improving respiratory symptoms. Thoracoplasty via anterior bony thorax resection was designed by Grillo for use in cervical exenteration and mediastinal tracheostomy [8, 9]. However, to best of our knowledge, only one previous report in the English-language literature has described the application of thoracoplasty to tracheal compression in which a partial sternectomy combined with brachiocephalic artery transection was performed [10]. Moreover, few Japanese studies have examined the partial resection of the anterior bony thorax to treat or prevent tracheobrachiocephalic fistula development [11]. In previous studies describing sternal tumors, reconstruction of the chest wall was required to prevent flail chest and respiratory impairment and to protect the underlying mediastinal structures after a complete or partial sternectomy [12]. In contrast, the need for chest wall reconstruction in patients with severe motor and intellectual disabilities is unclear, and sternal resection and chest wall reconstruction are extremely invasive.

The thoracoplastic procedure described as an inverse T-shaped sternotomy in the present report is a simple method that allows the sternum to be left open after a partial sternotomy for subsequent brachiocephalic artery transection surgery. We speculate that traction, rather than the gap between the halves of the divided manubrium, is important for improving mediastinal narrowing. Since the clavicle and the first rib remain attached to the halves of the divided manubrium, traction to the left or right of the manubrium can be maintained by laterally transecting the sternum. Neither patient showed postoperative chest fragility or any movement in the halves of the manubrium when moving the upper limbs. The advantages of the current procedure are (1) its simplicity; (2) avoidance of a sternotomy from the cranial side where vessels are thickly concentrated in a narrow area, carrying the risk of fatal vessel injury [13]; (3) good operative field for brachiocephalic artery transection (Fig. 1c); (4) possibility of shifting the position of the sternal end of the clavicle laterally (left or right); (5) minimal impairment of chest function and minimal deformation of the bony sternum, and (6) ability to distance the skin incision from the tracheostomy to reduce the risk of wound contamination. In fact, a wider cut surface of the sternum carries a potentially higher risk of osteomyelitis; however, if osteomyelitis occurs, treatment is relatively easy to administer because the bony sternum is open and the wound can be distanced from the tracheostomy.

The present study has some limitations. First, it did not examine whether thoracoplasty or brachiocephalic artery transection was superior in improving respiratory symptoms although the findings suggested that the latter may be superior. Although thoracoplasty may not be essential, the procedure described in the present study is easy to apply in most patients with tracheal stenosis requiring a brachiocephalic artery transection. Second, the effect of thoracoplasty alone was not able to be assessed. The thoracoplastic procedure described here can preserve the brachiocephalic artery, but because it is indicated for life-threatening respiratory events, brachiocephalic artery transection should be performed if there are no contraindications, such as anomalies of the circle of Willis.

## Conclusions

Our observations suggested that the inverse T-shaped sternotomy combined with brachiocephalic artery transection may relieve symptoms of tracheal stenosis due to severe chest deformation in patients with severe motor and intellectual disabilities. Nonetheless, our patients were followed for only 1 year; therefore, further research evaluating the long-term results of this procedure is warranted.

## Data Availability

The datasets used and/or analyzed during the current study are available from the corresponding author on reasonable request.

## Abbreviation

CT: Computed tomography
